# Functional Resilience and Response to a Dietary Additive (Kefir) in Models of Foregut and Hindgut Microbial Fermentation *In Vitro*

**DOI:** 10.3389/fmicb.2017.01194

**Published:** 2017-06-28

**Authors:** Gabriel de la Fuente, Eleanor Jones, Shann Jones, Charles J. Newbold

**Affiliations:** ^1^Departament de Ciència Animal, Escola Tècnica Superior d’Enginyeria Agrària, Universitat de LleidaLleida, Spain; ^2^Institute of Biological Environmental and Rural Sciences, Aberystwyth UniversityAberystwyth, United Kingdom; ^3^Chuckling Goat LtdLlandysul, United Kingdom

**Keywords:** microbial stability, digestive system, gut fermentation, kefir, *in vitro*

## Abstract

Stability in gut ecosystems is an important area of study that impacts on the use of additives and is related with several pathologies. Kefir is a fermented milk drink made with a consortium of yeast and bacteria as a fermentation starter, of which the use as additive in companion and livestock animals has increased in the last few years. To investigate the effect of kefir milk on foregut and hindgut digestive systems, an *in vitro* approach was followed. Either rumen fluid or horse fecal contents were used as a microbial inoculate and the inclusion of kefir (fresh, autoclaved, or pasteurized) was tested. Gas production over 72 h of incubation was recorded and pH, volatile fatty acids (VFAs), lactate and ammonia concentration as well as lactic acid (LAB) and acetic acid bacteria, and yeast total numbers were also measured. Both direct and indirect (by subtracting their respective blanks) effects were analyzed and a multivariate analysis was performed to compare foregut and hindgut fermentation models. Addition of kefir boosted the fermentation by increasing molar concentration of VFAs and ammonia and shifting the Acetate to Propionate ratio in both models but heat processing techniques like pasteurization or autoclaving influenced the way the kefir is fermented and reacts with the present microbiota. In terms of comparison between both models, the foregut model seems to be less affected by the inclusion of Kefir than the hindgut model. In terms of variability in the response, the hindgut model appeared to be more variable than the foregut model in the way that it reacted indirectly to the addition of different types of kefir.

## Introduction

Stability in gut microbial ecosystems is a trait based on the ability of a system to withstand change by minimizing perturbations through the ability of a complex microbiota to perform similar functions and, in time, return to a new equilibrium or the state before perturbation (McCann, 2000). It can be defined functional resilience as the way the system responds to the perturbation once it has already happened (Weimer, 2015). Thus, it should be measured through the performance of the community (in terms of metabolic activity and functioning) rather than the actual taxonomic composition and richness of that community. There is no apparent correlation between microbial diversity and stability, and thus the same microbial diversity does not necessarily imply equivalent stability (McCann, 2000). However, it has been postulated than species rich communities most likely host functionally similar species which can buffer against changes in species composition under changing environmental conditions (Dìaz and Cabido, 2001). Study of stability in gut environments is of vital importance to test potential effects of new additives in gut ecosystems, such as niche filling or niche replacement (Weimer, 2015). Moreover, a microbial population that is less stable can be more prone to disbiosis and colonization by opportunistic pathogens (Aziz et al., 2013). Stability can be considered in number of contexts including either; the stability of the microbial a population in terms of the number of microbes and their relative distribution or the functional resilience and response of an ecosystem in terms of fermentation outputs.

Digestive tract physiology greatly influences the microbial structure, through factors such as volume, retention time and absorption sites (Godoy-Vitorino et al., 2012). It has been suggested that in order to allow a longer retention time to ferment the fibrous material, foregut fermentative organs are more voluminous than hindgut fermentative organs (Stevens and Hume, 1995). Foregut formative organs undergo marked volume fluctuations because they tend to fill and empty, in contrast with hindgut organs that tend to be more constant in volume (Demment and Van Soest, 1985). The foregut contains a heterogeneous digesta composed mostly of plant solid material like cereal grains, leaves or entire plant parts from the diet, whereas the hindgut contents are made up of pre-digested plant material that in general presents a higher homogeneity (Stevens and Hume, 1995). Thus it is likely that these two models may respond differently to feed additives. One of the major differences between rumen and hindgut is associated to substrate quality available to fermentation by the microbial population in each site of digestive tract; in the rumen more high quality nutrients are available at a higher concentration than in the hindgut. Because of that, the concentration and diversity of microbial population in the rumen is higher than observed in the hindgut (Godoy-Vitorino et al., 2012).

Kefir is a slightly carbonated fermented beverage manufactured through the fermentation of milk with kefir starter grains (Prado et al., 2015). These grains are unique dairy starters that contain a symbiotic consortium of microorganisms strongly influenced by grain origin and culture conditions (Garrote et al., 2010). Although the total number of microorganisms and their relative composition in grains is variable and ill-defined (Prado et al., 2015), kefir grains are mainly composed of lactic acid bacteria (LAB) (*Lactobacillus* spp.), yeast (*Saccharomyces, Kluyveromyces, Kazachstania*, and *Lachancea* spp.), and on occasion acetic acid bacteria (AAB) (*Acetobacter* spp.) (Lopitz-Otsoa et al., 2006). Kefir contains proteins, lipids, and lactose together with ethanol and lactic acid. Kefir can be a useful nutritional source of calcium, essential amino acids, and vitamins (Otles and Cagindi, 2003). Some microbial strains belonging to the kefir consortia have been previously used as probiotics (Golowczyc et al., 2008) or as producers of antimicrobial compounds (Ryan et al., 1996; Rodrigues et al., 2005). To the authors’ knowledge, only few recent studies have been reported the use of milk kefir in either livestock or companion animals (Fouladgar et al., 2016), although it has been hypothesized that kefir might be a protein rich livestock feed for animals (Koutinas, 2003). Moreover, milk kefir composition includes some of the most commonly utilized probiotics in both ruminant and non-ruminant herbivores, such as yeast strains or *Lactobacillus* sp.

The probiotic effect of microorganisms can be direct (through the action of the microorganisms by themselves) or indirect (by the action of the metabolites that these microorganisms produce) (Vinderola et al., 2006). The most important metabolites produced in fermented milk products, like kefir, are likely to be peptides produced during fermentation (Yamamoto et al., 2003). It is generally assumed that fermented milk products must contain a viable microbial population to induce positive health effects. However, there are commercial advantages associated with the use non-viable microorganisms (longer shelf-life and better storage) (Vinderola et al., 2005). Pasteurization and autoclaving are widely used as heat processing techniques to reduce or eliminate bacterial load, and applied in a variety of situations, from clinical to nutritional purposes (Hossain et al., 2011; Pardo and Zufía, 2012). However, there is a thermal degradation of the nutrients associated to these processes that may affect any potentially probiotic effect. Autoclaving is generally considered a more severe technique than pasteurization, which is normally used to increase the shelf-life with little detrimental on the nutritional characteristics of the product.

*In vitro* gas production techniques are common used to evaluate feeds (Pell et al., 1998). Such techniques are simple to use, low in cost and increasingly being used to estimate microbial activity in the gut (Williams et al., 2000), to evaluate toxicity of secondary compounds (Ammar et al., 2004) and to screen the effect of feed additives on gut fermentation (Colombatto et al., 2003).

The aim of this study was to assess *in vitro* the functional resilience, as a measure of stability, of two different gut models representing foregut and hindgut fermenters. To achieve that objective, kefir produced from goats milk was used to provoke a significant perturbation in the studied microbial models. Moreover, two heat processing techniques were also applied to the kefir to study different sources of perturbation, including live v dead microorganisms.

## Materials and Methods

### Ethics Statement

All animal procedures were carried out according to the Animals (Scientific Procedures) Act 1986 (PLL 40/3653; PIL 40/9798) in accordance with the guidelines of the European Directive 2010/63/EU and after approval by the Aberystwyth University’s Internal Ethical Review Panel.

### Foregut Model

Rumen fluid was collected separately from four dairy cows fed a diet composed of perennial ryegrass hay and concentrate at 67:33 on DM basis via permanently established rumen cannulae, and strained through a double layer of muslin and stored under CO_2_ at 39°C (see Table S1 in Supplementary Material for chemical composition of experimental diets). Rumen fluid was diluted 2:1 with incubation solution (Theodorou et al., 1994) under CO_2_ and 50 mL of buffered rumen fluid was incubated with 500 mg of a mixed diet (30:70 ground barley/alfalfa mix ground to pass through a 1 mm^2^ sieve). General batch incubation procedures were all performed following (Belanche et al., 2016).

### Hindgut Model

Fresh fecal material was collected separately from four horses the morning of the experiment, kept warm and mixed with buffer solution (Theodorou et al., 1994) under CO_2_ at 39°C (1:2 w/v). Then, the mix was strained through a double layer of muslin and 50 mL was incubated with 500 mg of a pre-digested substrate as described below.

#### Pre-digestion of the Substrate

Pre-digestion of the dietary substrate (30:70 ground barley/alfalfa mix ground to pass through a 1 mm^2^ sieve) used in the hindgut model was performed following a double digestion in Pepsin HCl and Pancreatin (Calsamiglia and Stern, 1995). Pepsin HCl digestion was carried out using 10 g of feed in a pre-weighed 45 μm^2^ Dacron bag. Bags containing substrate were submerged in 1600 mL of a solution of 2 g/L pepsin in 0.075 M HCl and incubated for 30 min at 38°C. Then, bags were transferred into a second container of flowing clean water for 10 min. Pancreatin digestion was then done by submerging bags in 1600 mL of a solution of 0.1% Pancreatin (Sigma P1500) in water (pH 8). Bags were incubated for 60 min at 38°C and transferred to a second container of flowing clean water for 10 min. Finally, substrate was oven dried at 50°C overnight and reweighed.

### Preparation of Kefir

Unflavored kefir was produced by The Chuckling Goat LTD (Llandysul, Wales, SA44 6DS) from raw goat milk containing 2.9% fat. Samples were kept on ice until use. Samples (500 mL) of kefir to be pasteurized were aseptically transferred to a glass Erlenmeyer flask (2 L), heated for 30 min at 62.5°C and then cooled rapidly in water and ice. Samples (500 mL) of kefir to be autoclaved were aseptically transferred to a glass Erlenmeyer flask (2 L), heated for 20 min at 121°C and then cooled rapidly in water and ice.

### Incubation and Sampling

**FIGURE 1:**
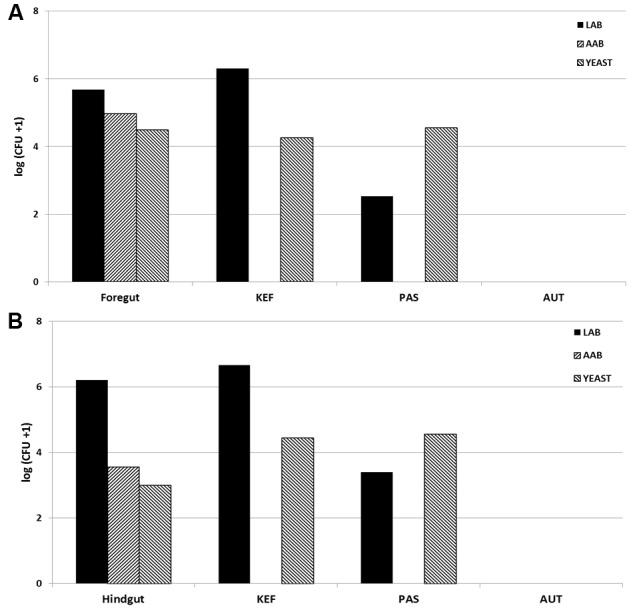
Initial concentration of Lactic acid bacteria (LAB), Acetic acid bacteria (AAB) and yeast [log (CFU +1) per mL] in rumen fluid **(A)** or horse fecal material **(B)**, and in Kefir (KEF, unaltered kefir; PAS, pasteurized kefir; AUT, autoclaved kefir).

All incubations were run in duplicate for each animal. Kefir was added at a concentration of 10 mL per L of incubation (0.5 mL per bottle) prior to the addition of buffered rumen fluid or equine digesta, according to the following treatments: CTR (no added kefir), KEF (unaltered kefir), AUT (autoclaved kefir), and PAS (pasteurized kefir). All incubations were in 120 mL Wheaton bottles under CO_2_ sealed with an airtight seal, and incubated at 39°C. Blanks (without substrate) of the four treatments (CTR, KEF, AUT, and PAS) were also included in the incubation (a summary of the treatments in shown in **Table 1**). Samples of buffered rumen/horse fluid at inoculation and kefir used as inoculate were retained to determine initial LAB, AAB, and yeast levels (see **Figure 1**) as explained below.

**Table 1:** Treatments applied in the experiment (BLK, blank; pkefir, pasteurized kefir; akefir, autoclaved kefir).

Additive	I^∗^	I+S^∗^	(I+S)-I^∗^
No additive	BLK	CTR	CTRB
Unaltered kefir	kefir + BLK	KEF	KEFB
Pasteurized kefir	pkefir + BLK	PAS	PASB
Autoclaved kefir	akefir + BLK	AUT	AUTB


*^∗^I, inoculate; I+S, inoculate plus substrate; (I+S)-I, treatments subtracting their respective blanks.*

Gas production was measured at 2, 4, 6, 8, 12, 24, 48, and 72 h of incubation using a TP704 Manometer (DELTA OHM, Italy). To convert pressure to volume we used a linear regression between pressure and known air volumes at standard incubation temperatures. After 24 h of incubation a sample (10% v/v) of liquid incubation media was retained for determining volatile fatty acid (VFA) (1.6 mL of sample in 0.4 mL of 20% orthophosphoric containing 20 mM 2-ethylbutric acid as internal standard), ammonia (0.8 mL of sample in 0.2 mL of 25% TCA) by gas liquid chromatography (Jouany, 1982) and a phenol hypochlorite method (Weatherburn, 1967), respectively; pH was also recorded after 24 h incubation. Concentrations of total lactate were measured using the EnzytecTM D/L-Lactic Acid kit (r-biopharm Rhone, Ltd, Glasgow).

Lactic acid bacteria counts from samples after 24 h of incubation were performed on MRS medium (pH 6.5) from Thermo Fisher (De Man et al., 1960) and incubated at 30°C for 3 days. Plates were overlaid to create a microaerophilic environment. To inhibit yeast growth pimaricin (100 mg/l) was added to the media. Yeasts and molds were grown on OGYE medium (Oxytetracycline-Glucose-Yeast Extract Agar, pH 7.6; Thermo Fisher), supplemented with Oxytetracycline GYE Selective supplement and incubated at 25°C for 7 days. Acetic acid bacterial were counted on the medium described by Guillamón (2000) containing 5% glucose, 1% yeast extract, and 2% agar. To inhibit yeast and LAB growth pimaricin (100 mg/l) and penicillin (3 μg/mL) were added to the media. Plates were overlaid to create a microaerophilic environment and incubated at 25°C for 2 days.

### Gas Production Curve Fitting

An exponential model was used (Ørskov and McDonald, 1979) to describe the kinetics of the accumulated gas production profiles:

$$Y=a+b\left(1-e^{\left(-ct\right)}\right)$$

Where “Y” is the accumulated gas produced over time in mL; “t” is time in hours; **“a”** and **“b”** parameters are used to explain the potential of fermentation (“a + b” reflects the maximum potential of fermentation), and **“c”** parameter is the gas production rate constant, used to explain the speed of fermentation.

### Statistical Analysis

Kefir extracts effects were analyzed with JMP Pro 11 (SAS, 2013) following a one-way ANOVA, considering number of bottles as replicates and “treatment” as main factor. Moreover, to evaluate the indirect influence of kefir on the microbial population in a second analysis, values observed in the blanks were subtracted to each treatment and analyzed again following a one-way ANOVA. Means were compared with a Tukey test at *P* < 0.05. P between 0.1 and 0.05 were considered trends.

Multivariate analyses, including Permutational Analysis of variance (Adonis) and Canonical Correspondence Analysis, were performed using the package “vegan” from the R statistical program (R Core Team, 2014); cluster analysis and analysis of the dispersion of the multivariate data were conducted calculating the similarity distances by the “Bray–Curtis” method, also from the package “vegan” in R. Red circumferences were displayed assuming a confidence interval of 95%.

## Results

Analysis of fermentation parameters and kinetics in the foregut model, as well as the concentration of LAB, AAB, and yeast are presented in **Table 2** and Supplementary Table S2. Addition of kefir prepared in different ways (autoclaved, pasteurized, or unaltered) caused an increase in the concentration of total VFAs after 24 h of incubation, compared to the control (*P* < 0.05). Molar proportion of acetate decreased when kefir was added, regardless of the treatment. The molar proportions of propionate and n-butyrate also increased when kefir was present in the incubations. Concentration of ammonia was also higher in bottles incubated with kefir (KEF, AUT, and PAS) compared to the control. KEF bottles had similar lactate concentration than control, and lower than PAS bottles (*P* < 0.05). Kefir associated bacteria (LAB and AAB) and yeast concentrations didn’t differ from control after 24 h of incubation, although a numerical increase was found in AAB and yeast; both pH and fermentation potential (measured as “a+b”) were altered when any of the kefir treatments were applied. Heat processing of kefir also had influence on the fermentation, having PAS bottles a higher molar concentration of VFAs and a lower molar proportion of acetate (*P* < 0.05).

**Table 2:** Fermentation parameters after 24 h of incubation of a mixed diet in a foregut model of rumen fermentation incubated with kefir (CTR), unaltered kefir (KEF), autoclaved kefir (AUT), or pasteurized kefir (PAS).

	CTR	KEF	AUT	PAS	SED	Significance
**Fermentation products**						
Total VFA (mM)	60.0^c^	63.2^ab^	61.8^b^	64.4^a^	0.793	^∗∗^
Acetate (%)	62.1^a^	60.2^c^	60.5^b^	60.2^c^	0.096	^∗∗∗^
Propionate (%)	21.3^b^	21.5^a^	21.6^a^	21.6^a^	0.058	^∗∗^
*N*-Butyrate (%)	1.02^b^	1.16^a^	1.15^a^	1.14^a^	0.023	^∗∗∗^
C2:C3 ratio	2.91^a^	2.80^b^	2.81^b^	2.79^b^	0.010	^∗∗∗^
NH_3_-N (mM)	8.36^b^	10.23^a^	10.29^a^	10.14^a^	0.364	^∗∗∗^
Lactate (mM)	0.38^c^	0.63^bc^	0.86^ab^	0.94^a^	0.150	^∗^
**Microbiology (log CFU +1)**						
LAB^†^	5.74	5.38	5.37	5.52	0.176	NS
AAB^†^	3.24	3.52	3.37	3.67	0.180	NS
Yeast	2.89	2.94	2.94	2.93	0.061	NS
**Fermentation parameters**						
pH	6.61^a^	6.52^b^	6.48^b^	6.48^b^	0.039	^∗^
c^¥^	0.05	0.06	0.06	0.06	0.003	NS
a+b^∓^	96.2^b^	106.6^a^	109.1^a^	108.2^a^	3.691	^∗^


*SED means Standard error of the difference between means (*N* = 4). ^∗^*P* < 0.05; ^∗∗^*P* < 0.01; ^∗∗∗^*P* < 0.001; NS means *P* > 0.1. †LAB, lactic acid bacteria; AAB, acid acetic bacteria. ^¥^Gas production rate, according to the following formula: Y = a + b (1 - e^*(–ct)*^). ^∓^Maximum potential of fermentation, expressed in mL of gas per gr of substrate. Within a row, means without a common superscript differ (*P* < 0.05).*

**FIGURE 2:**
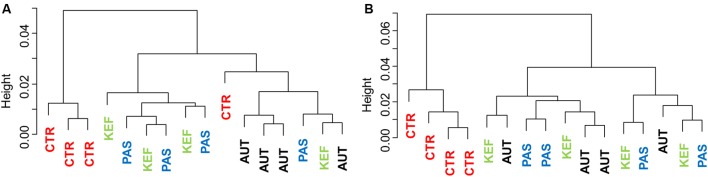
Dendrogram using the Bray–Curtis distances of foregut **(A)** or hindgut **(B)** model fermentation parameters (molar concentrations of Ammonia, Acetate, Propionate, *N*-butyrate, branched-chained VFA and Lactate, log concentrations of LAB, AAB and yeast, pH and coefficients “a+b” and “c”) after 24 h of incubation of a mixed diet incubated with kefir (CTR), unaltered kefir (KEF), autoclaved kefir (AUT), or pasteurized kefir (PAS) (*N* = 4).

**FIGURE 3:**
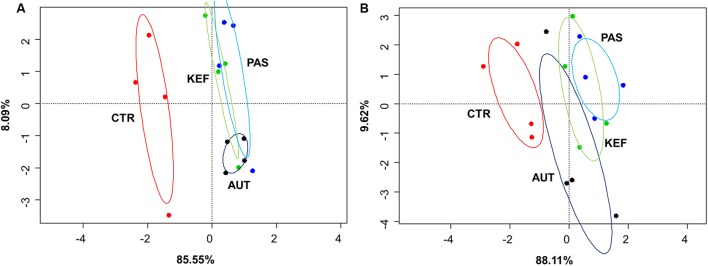
Canonical correspondence analysis of foregut **(A)** or hindgut **(B)** model fermentation parameters [molar concentrations of Ammonia, Acetate, Propionate, *N*-butyrate, branched-chained VFA and Lactate, log concentrations of LAB, AAB and yeast, pH and coefficient’s “a+b” (potential of fermentation) and “c” (speed of fermentation)] after 24 h of incubation of a mixed diet incubated with kefir (CTR), unaltered kefir (KEF), autoclaved kefir (AUT), or pasteurized kefir (PAS) (*N* = 4). Colored circles show the confidence interval of each group at a 95% level.

Dendrograms created with the pairwise distances of the all fermentation data sets (**Figure 2A**), clustered as three main groups, defined by CTR, AUT and a third mixed group with KEF and PAS. Permutational Analysis of Variance also indicated a significant influence of the studied treatments on the fermentation (*P* = 0.0004). These results were then confirmed in the CCA plot (**Figure 3A**) in which CTR samples separated from the other three groups.

As with the foregut model, most of metabolites analyzed from the hindgut model were present at higher concentrations in bottles incubated with any kind of kefir (*P* < 0.05, **Table 3** and Supplementary Table S3). The inclusion of kefir in the incubation in all its types induced a significant shift in most of the fermentation parameters studied including total VFA concentration, molar proportion of acetate, propionate, butyrate as well as ammonia, and the ratio of C2:C3 (*P* < 0.05). The type of heat processing influenced the results, with AUT giving rise to lower total VFA and ammonia production than the PAS treatment. No changes were observed in lactate concentration or in LAB, AAB, and yeast numbers when they were compared with the control (see **Table 3**). In terms of fermentation kinetics, only the maximum potential of fermentation (coefficient “a+b”) increased with the inclusion of any type of kefir (*P* < 0.01).

**Table 3:** Fermentation parameters after 24 h of incubation of a mixed diet in a hindgut model of equine fermentation incubated with kefir (CTR), unaltered kefir (KEF), autoclaved kefir (AUT), or pasteurized kefir (PAS) after 24 h of incubation.

	CTR	KEF	AUT	PAS	SED	Significance
**Fermentation products**						
Total VFA (Mm)	35.3^c^	40.6^ab^	39.7^b^	42.0^a^	0.725	^∗∗∗^
Acetate (%)	60.0^a^	58.8^b^	58.8^b^	58.4^b^	0.352	^∗∗^
Propionate (%)	26.6^b^	27.8^a^	27.6^a^	27.6^a^	0.236	^∗∗^
*N*-Butyrate (%)	1.98	1.99	2.06	2.12	0.115	NS
C2:C3 ratio	2.25^a^	2.11^b^	2.14^b^	2.12^b^	0.021	^∗∗∗^
NH_3_-N (mM)	14.2^c^	15.7^ab^	14.7^bc^	16.0^a^	0.572	^∗^
Lactate (mM)	2.34	2.46	2.25	2.42	0.232	NS
**Microbiology (log CFU +1)**						
LAB^†^	7.49	7.60	7.69	7.63	0.122	NS
AAB^†^	6.60	6.61	6.73	6.73	0.143	NS
Yeast	2.66	2.59	2.76	2.38	0.184	NS
**Fermentation parameters**						
pH	6.54	6.54	6.52	6.56	0.037	NS
c^¥^	0.05	0.05	0.06	0.05	0.003	NS
a+b^∓^	54.0^b^	65.7^a^	64.9^a^	68.3^a^	2.961	^∗∗^


*SED means Standard error of the difference between means (*N* = 4). ^∗^*P* < 0.05; ^∗∗^*P* < 0.01; ^∗∗∗^*P* < 0.001; NS means *P* > 0.1. †LAB, lactic acid bacteria; AAB, acid acetic bacteria. ^¥^Gas production rate, according to the following formula: Y = a + b (1 - e^*(–ct)*^). ^∓^Maximum potential of fermentation, expressed in mL of gas per gr of substrate. Within a row, means without a common superscript differ (*P* < 0.05).*

Multivariate analysis (**Figures 2B**, **3B**), indicated that CTR samples grouped together with no clear separation observed between the kefir based treatments. Permutational Analysis of Variance indicated a treatment effect (*P* = 0.003), supporting the grouping between CTR and kefir treatments seen in the dendrogram. Pairwise comparisons between treatments (**Table 6**) also indicated that CTR samples presented a distinctive profile, compared with the rest of the treatments.

### Differential Effect

**FIGURE 4:**
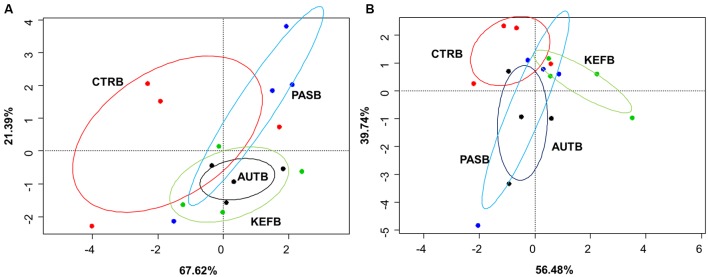
Canonical correspondence analysis of foregut **(A)** or hindgut **(B)** model fermentation parameters [molar concentrations of Ammonia, Acetic, Propionic, *N*-butyrate, branched-chained VFA and Lactic acids, log concentrations of LAB, AAB and yeast, pH and coefficients “a+b” (potential of fermentation) and “c” (speed of fermentation)] after 24 h of incubation of a mixed diet incubated with kefir (CTRB), unaltered kefir (KEFB), autoclaved kefir (AUTB), or pasteurized kefir (PASB) after subtracting their respective blanks (containing only kefir), after 24 h of incubation (*N* = 4). Colored circles show the confidence interval of each group at a 95% level.

**FIGURE 5:**
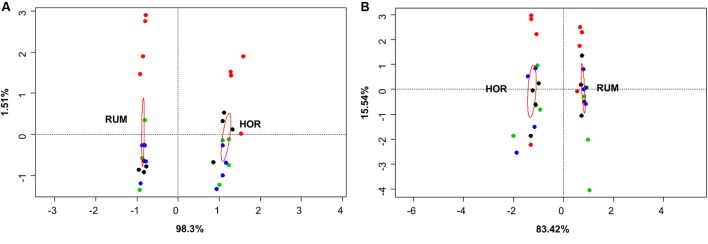
Canonical correspondence analysis of both fermentation models using direct values of fermentation parameters [molar concentrations of Ammonia, Acetate, Propionate, *N*-butyrate, branched-chained VFA and Lactate, log concentrations of LAB, AAB and yeast, pH and coefficients “a+b” (potential of fermentation) and “c” (speed of fermentation)] **(A)** or after subtracting their respective blanks (containing only kefir) **(B)**. RUM and HOR correspond to foregut and hindgut models, respectively. Red circles show the confidence interval of each group at a 95% level. Plot colors indicate the treatment [**A**: CTR KEF, PAS, and AUT; **B**: CTRB, KEFB, PASB (blue) and AUTB (black)].

Results obtained after subtracting the specific blank to the respective incubation are summarized in **Tables 4**, **5**, **7**, Supplementary Tables S4, S5, and **Figures 4**, **5B**. A complete definition of terms CTRB, PASB, AUTB, and KEFB is explained in **Table 1**. The most obvious differences observed in the foregut model were the alteration in the acetate and propionate proportions, and thus the C2:C3 ratio (**Table 4**). Molar proportion of propionate increased more in CTRB than in kefir-based treatments. Moreover, the increase of molar proportion of acetate was higher in PASB than in AUTB and KEFB (*P* < 0.05). Neither ammonia nor lactate concentrations were modified by the kefir-based treatments compared to the control (*P* > 0.05).

**Table 4:** Fermentation parameters after 24 h of incubation of a mixed diet in a foregut model of rumen fermentation incubated with kefir (CTRB), unaltered kefir (KEFB), autoclaved kefir (AUTB), or pasteurized kefir (PASB) after subtracting their respective blanks (containing only kefir), after 24 h of incubation.

	CTRB	KEFB	AUTB	PASB	SED	Significance
**Fermentation products**						
Total VFA (Mm)	17.4	18.1	17.9	18.1	0.509	NS
Acetate (%)	-0.58^c^	0.72^b^	0.44^b^	1.59^a^	0.400	^∗∗^
Propionate (%)	7.43^a^	5.48^b^	5.45^b^	5.56^b^	0.451	^∗∗^
*N*-Butyrate (%)	-1.05	-0.81	-0.73	-0.82	0.165	NS
C2:C3 ratio	-0.90^b^	-0.49^a^	-0.51^a^	-0.46^a^	0.039	^∗∗∗^
NH_3_-N (mM)	-2.71	-2.09	-1.88	-2.97	0.876	NS
Lactate (mM)	-0.26	-0.51	-0.43	-0.71	0.344	NS
**Microbiology (log CFU +1)**						
LAB^†^	0.30	0.09	0.09	0.07	0.142	NS
AAB^†^	0.16	0.22	0.35	0.17	0.151	NS
Yeast	0.00	-0.12	0.14	-0.14	0.271	NS
**Fermentation parameters**						
pH	-0.22	-0.19	-0.18	-0.14	0.068	NS
c^¥^	0.008^a^	0.005^ab^	0.004^bc^	0.002^c^	0.0021	T
a+b^∓^	55.8	59.8	59.6	60.7	3.129	NS


*SED means Standard error of the difference between means (*N* = 4). T means 0.1 > *P* > 0.05; ^∗∗^*P* < 0.01; ^∗∗∗^*P* < 0.001; NS means *P* > 0.1. †LAB, lactic acid bacteria; AAB, acid acetic bacteria. ^¥^Gas production rate, according to the following formula: Y = a + b (1 - e^*(–ct)*^). ^∓^Maximum potential of fermentation, expressed in mL of gas per gr of substrate. Within a row, means without a common superscript differ (*P* < 0.05).*

**Table 5:** Fermentation parameters after 24 h of incubation of a mixed diet in a hindgut model of equine fermentation incubated with kefir (CTRB), unaltered kefir (KEFB), autoclaved kefir (AUTB), or pasteurized kefir (PASB) after subtracting their respective blanks (containing only kefir), after 24 h of incubation.

	CTRB	KEFB	AUTB	PASB	SED	Significance
**Fermentation products**						
Total VFA (Mm)	28.2^a^	26.6^b^	25.9^b^	27.3^ab^	0.754	T
Acetate (%)	0.13^ab^	-0.01^ab^	0.27^a^	-0.15^b^	0.151	T
Propionate (%)	1.64	1.68	1.81	1.952	0.331	NS
*N*-Butyrate (%)	-0.29	-0.30	-0.30	-0.29	0.065	NS
C2:C3 ratio	-0.24	-0.24	-0.24	-0.29	0.058	NS
NH_3_-N (mM)	-0.92	-1.00	-0.93	-0.86	0.400	NS
Lactate (mM)	-0.01	-0.07	0.27	0.22	0.201	NS
**Microbiology (log CFU +1)**						
LAB^†^	0.46^a^	-0.13^b^	0.16^ab^	0.19^ab^	0.182	^∗^
AAB^†^	-0.42	-0.11	-0.47	-0.77	0.284	NS
Yeast	-0.05	-0.01	-0.03	-0.09	0.055	NS
**Fermentation parameters**						
pH	-0.19^a^	-0.30^bc^	-0.22^ab^	-0.32^c^	0.041	^∗^
c^¥^	-0.02^a^	-0.05^c^	-0.03^b^	-0.04^bc^	0.004	^∗∗∗^
a+b^∓^	98.7	95.0	96.7	95.7	3.808	NS


*SED means Standard error of the difference between means (*N* = 4). T means 0.1 > *P* > 0.05; ^∗^*P* < 0.05; ^∗∗∗^*P* < 0.001; NS means *P* > 0.1. †LAB, lactic acid bacteria; AAB, acid acetic bacteria. ^¥^Gas production rate, according to the following formula: Y = a + b (1 - e^*(–ct)*^). ^∓^Maximum potential of fermentation, expressed in mL of gas per gr of substrate. Within a row, means without a common superscript differ (*P* < 0.05).*

**Table 6:** Pairwise distances between treatments, after 24 h of incubation in the foregut (RUM) or hindgut (HOR) model incubated with kefir (CTR), unaltered kefir (KEF), autoclaved kefir (AUT), or pasteurized kefir (PAS).

	Rumen			Horse		
		
Pairs	Distance	F-Model	*P*-Adjusted	Distance	F-Model	*P*-Adjusted
CTR vs. KEF	4.55	8.55	0.116	7.16	19.88	0.003
CTR vs. AUT	4.77	15.13	0.003	6.34	14.19	0.003
CTR vs. PAS	5.11	13.14	0.003	8.58	33.44	0.058
KEF vs. AUT	2.05	0.56	0.658	2.93	0.54	0.572
KEF vs. PAS	2.36	0.66	0.608	2.89	0.94	0.499
AUT vs. PAS	2.11	1.47	0.392	3.53	2.41	0.339


*Permutational analysis of variance was performed using Bray–Curtis distances measurements of fermentation parameters data. Higher Pseudo-F and higher similarities and lower *P*-values correspond to greater differences in the fermentation (*n* = 4).*

**Table 7:** Pairwise distances between treatments after 24 h of incubation in the foregut (RUM) or hindgut (HOR) model incubated with kefir (CTRB), unaltered kefir (KEFB), autoclaved kefir (AUTB), or pasteurized kefir (PASB) after subtracting their respective blanks (containing only kefir).

	RUM			HOR		
		
Pairs	Distance	F-Model	P-Adjusted	Distance	F-Model	*P*-Adjusted
CTRB vs. KEFB	7.03	1.47	0.696	4.19	1.37	0.676
CTRB vs. AUTB	5.72	1.37	0.696	3.77	1.44	0.676
CTRB vs. PASB	6.56	2.59	0.528	4.20	0.98	0.676
KEFB vs. AUTB	6.05	0.04	0.942	3.37	0.36	0.779
KEFB vs. PASB	6.21	0.22	0.942	4.00	0.38	0.779
AUTB vs. PASB	5.66	0.45	0.942	3.61	0.56	0.779


*Permutational analysis of variance was performed using Bray–Curtis distances measurements of fermentation parameters data. Higher Pseudo-F and higher similarities and lower *P*-values correspond to greater differences in the fermentation (*n* = 4).*

Permutational ANOVA applied to the dataset indicated that samples did not group separately (*P* = 0.513), most probably due to the higher variability observed within samples from the same treatment (**Figure 4A**). The same effect was then confirmed in the pairwise comparison (**Table 7**), in which no treatment showed statistical differences between them.

Results obtained in the hindgut model also followed a similar trend to those from the foregut model, in which no major differences were observed between treatment, apart from a decrease in pH (*P* < 0.05) in CTRB samples and a decrease in the speed of fermentation (coefficient “c”) in kefir-based treatments.

Permutational ANOVA indicated no differences due to treatment (*P* = 0.572), confirming the pairwise comparison among treatments (**Table 7**).

**FIGURE 6:**
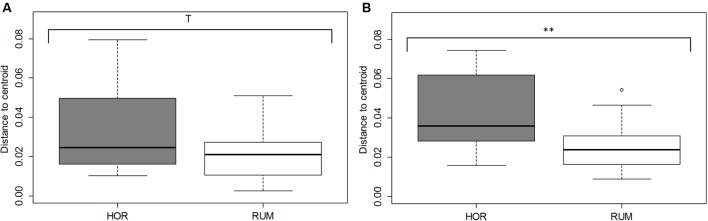
Dispersion of fermentation parameter data [molar concentrations of Ammonia, Acetate, Propionate, *N*-butyrate, branched-chained VFA and Lactate, log concentrations of LAB, AAB and yeast, pH and coefficients “a+b” (potential of fermentation) and “c” (speed of fermentation)], measured as distance form their corresponding centroid. Bray–Curtis method was used to generate the distances between samples. RUM and HOR correspond to foregut and hindgut models, respectively. Figures were obtained from multivariate data from direct values **(A)** or after subtracting their respective blanks **(B)**. T means 0.1 > *P* > 0.05; ^∗∗^*P* < 0.01.

Comparative analysis between both fermentative models (Foregut vs. hindgut) is shown in **Figures 5**, **6**. Multivariate analysis showed a clear fermentation model effect (*P* < 0.001) in both direct (**Figure 5A**) and differential (**Figure 5B**) analysis. Moreover, to study the variance in response from the two models (i.e., dispersion), a simple ANOVA was performed on the data generated by the distance to the centroids in the two fermentative models (see **Figure 3B**). As can be seen in **Figure 5**, both models grouped separately for both situations (direct effect and “differential” effect), and the dispersion (measured as the distance to the centroid) in the hindgut model was higher in both situations, becoming significant when the “differential” effect was studied (**Figure 6B**, *P* = 0.009).

## Discussion

### Overall Effect of Kefir

The effect of kefir on digestive systems has been described by several authors in terms of anti-inflammatory, anti-allergic (Lee et al., 2007) and probiotic effect (Farnworth, 2006). It is known that kefir can interact with gut microbiota (Carasi et al., 2015) and control pathogenic infections such as *Clostridium difficile* (Bolla et al., 2013). In this study we investigated the action of kefir using two gut models: a foregut model (RUM) that represents ruminant fermentation, and hindgut model (HOR), that represents the non-ruminant herbivore species. To achieve this objective, we used rumen fluid as an inoculate for the foregut model and horse fecal material for the hindgut model. The microbial community present in both systems is known to behave differently in terms of the nutritional ecology of the host (Van Soest, 1994). The rumen has a major role in stabilizing host nutrition (Newbold et al., 2001) and its capability as detoxifier has been reviewed (Upadhaya et al., 2010). On the other hand, horse hindgut microbiota is highly sensitive to diet changes and certain diet related pathologies, like lameness have been associated with a lower core microbial population in the hindgut (Steelman et al., 2012). This study aimed to use the kefir as an agent to challenge these two distinctive models (one robust and other fragile) and induce an alteration in the fermentation pattern. The dose used in this study was chosen based on preliminary results obtained in similar incubation conditions, tested in both fermentation models and following a dose-response analysis (data not shown). No predigestion of kefir was performed in the hindgut model, because the objective of this study was to observe the effect of the same type of disturbance in both fermentation models, and hence, the predigestion of kefir would indubitably have modified the original properties of this additive. In order to apply this knowledge in practical situations, it would be needed to include a dose-response study at *in vivo* conditions, since the feeding of kefir would imply its digestion prior to reaching the hindgut.

Kefir changed the fermentation pattern in our foregut model (**Table 2**) in terms of metabolite concentration, C2:C3 ratio and the potential extent of fermentation (“a+b” parameter). These results can be explained by the metabolite composition of kefir and its probiotic effect, which could have provided extra sources of energy and protein to the microbiota (Farnworth, 2006). The effects are probably due to the combination of both microflora in the kefir and its ingredients/nutrients that boost the fermentation. In general, a greater increase compared to the control in most of the analyzed metabolites was observed when pasteurized kefir was used (**Table 2**), suggesting that most of this effect may have been caused by the nutrients provided by the kefir and not by the probiotic microflora. Most of metabolites concentrations did not differ between pasteurized and untreated kefir. Autoclaved kefir resulted in a lower concentration of metabolites compared to the other two sources of kefir. Autoclaving is an aggressive method of sterilization that can lead to protein denaturalization and formation of poorly fermentable material (Parsons et al., 1992). It seems that part of the extra substrate provided by the kefir became unavailable after its sterilization by autoclaving.

A similar pattern was observed in the hindgut model, in which most of the major metabolites increased when kefir was included in the incubation. Moreover, a shift toward propionate fermentation occurred, resulting in higher proportions of propionate and lower of acetate and C2:C3 ratio when compared to the control. A higher concentration of LAB in those incubations could potentially have promoted an increase in the production of propionate, and hence a shift in the fermentation pattern.

It’s noticeable than both lactic and AAB concentrations in PAS treatment were numerically higher than in KEF, suggesting that after 24 h bacteria belonged to the kefir inoculum did not survive or at least reached an equilibrium with the microbiota present at the incubations. Moreover, a higher concentration of fermentable metabolites and in particular lactic acid could have promoted a lower pH and a change in the fermentation pattern. Pearson’s negative correlations between lactate concentration and either pH or C2:C3 ratio confirmed this process (*r* = -0.594, *P* = 0.01 for pH and *r* = -0.662, *P* = 0.005 for C2:C3 ratio). Although it appears that positive effects are promoted by Kefir in rumen and hindgut models, long term studies are needed to evaluate the possible transience of kefir effects over time on gut microbial communities.

### Differential Effect

In this study we present two types of information in each of the two gut models. First (**Tables 2**, **3**) we tried to assess the overall effect of the inclusion of kefir, either unaltered or after autoclaving or pasteurization on the fermentation pattern in both foregut and hindgut models. Secondly, we aimed to assess the indirect effect of kefir on the microbial population, by studying the changes in the fermentation of the substrate when incubated with kefir. This “differential” effect was calculated by subtracting the values obtained in the specific blanks from the correspondent treatments (**Table 1**). Thus, differences among the treatments in the values obtained after subtracting their respective blanks could indicate any effect not directly related to fermentation of the kefir but indirectly via effects on microbial activity (**Tables 4**, **5**). This is a novel approach that could potentially identify changes in the microbial structure. However, some of the results must be interpreted with care, since efficiency of utilization of a compound can change in presence of additional substrates (Hall and Weimer, 2007; Rezaii et al., 2010), and with this approach we are assuming a similar efficiency of utilization between samples.

In general no major differences were observed after including this correction, apart from changes in the molar proportions of acetic and propionic (*P* < 0.01). The production of total VFA was also numerically higher in kefir based treatments and in the potential of fermentation (“a+b”), suggesting a more efficient microbiota in using fermentable material. However, the high variability made impossible to find statistical differences between treatments.

The differential approach performed in the hindgut model showed even less significant differences than those observed in the foregut model (**Table 5**). Total VFA production was affected in the opposite way to that observed in the foregut model (*P* < 0.1), suggesting that inclusion of pasteurized kefir can indirectly reduce the efficiency of substrate utilization. The pH decrease due to the substrate fermentation was more acute in bottles with either live or pasteurized kefir; moreover, the speed of fermentation was lower in those cases, confirming previous studies showing an inverse relation between pH and rate of fermentation (Grant and Mertens, 1992).

### Hindgut vs. Foregut Fermentation Models

Composition of LAB, AAB, and yeast in both foregut and hindgut models differed (see **Figure 1**), presenting the latter a higher (numerical) concentration of LAB and a lower of AAB and yeast. Viable microorganisms in kefir inoculates included a major contribution of LAB and yeast. Pasteurization seemed to affect mostly LAB but not yeast numbers, and autoclaving eliminated all the viable bacteria. In this scenario, it was hypothesized that kefir in their different presentations would affect in a different way the foregut and the hindgut systems. However, after 24 h of incubation no differences were observed in microorganisms concentrations (*P* > 0.1 in both models, **Tables 2**, **3**), indicating the capability of both models to react to the perturbation induced (inclusion of kefir in different formats) and to get to a new equilibrium. Resilience is generally higher in species rich environments, but the disturbance plays out in two different organizational levels: changing biodiversity can have a strong influence on the community level, but the effect on the ecosystem not necessarily is evident (Liebergesell et al., 2016).

Inclusion of kefir seemed to exert a more pronounced effect in the foregut model, increasing the concentration of most of the measured metabolites. Only with total VFA did kefir addition induce a higher change in the hindgut model. Microbial cell synthesis depends on the availability of precursors (such as simple sugars, nucleic acids, ammonia, or minerals). If bacteria are limited through energy source, total energy and growth utilization can be explained by growth and maintenance functions of the microorganisms (Russell and Cook, 1995; Russell, 2007). However, if growth is not limited by energy but by other nutrients (e.g., nitrogen), energetic uncoupling occurs, and bacteria can spill ATP in reactions that cannot be categorized as maintenance *per se* (Russell and Cook, 1995; Russell, 2007). In the hindgut, the substrate that reaches the hindgut highly depends on pre-cecal digestibility of feeds, which means that cytoplasmic protein (nitrogen) and soluble sugars reach the hindgut in low amounts. However, the majority of cell wall carbohydrates and linked nitrogen will reach the hindgut since limited hydrolysis of these constituents occurs either in the stomach or in the pre-cecal environment. If we consider the type of substrate that gets to the hindgut, we can expect it to be nitrogen limiting, and a situation of energetic uncoupling seems feasible. The hindgut environment would aim to favor VFAs production by increasing microbial metabolism without net microbial growth. In this study, it was observed an increase of 15.5% in the production of VFAs, compared with that observed in the foregut (5.2%). However, the increase of ammonia was only of 8.9%, indicating a lower activity related with the nitrogen catabolism and cell growth. As the substrate that reaches the hindgut will be nitrogen limiting, the environment should promote microbial activity but without net growth thus favoring VFAs production (Russell, 2007; Santos et al., 2011).

Both host diet (Tajima et al., 2001) and the relationships among groups of organisms can influence gut bacterial diversity (Ley et al., 2008), based on fecal microbial composition, found that foregut and hindgut herbivores fermenters grouped separately. Differences between foregut and hindgut environments include different viscosity due to the water content (Hecker and Grovum, 1971), pH (bile and bicarbonate buffering) (Mackie and Wilkins, 1988), particle size and VFA concentration fluctuations (Sato and Shiogama, 2010). Microbial communities altered by disturbances do not return to their original composition for after some time (Allison and Martiny, 2008). However even if the microbial composition may change, the new community that appears after the disturbance might be similar to the original in terms of metabolic activity (Weimer, 2015). Considering this, expression of changes (for instance, by shifts in the fermentation pattern) can indicate a more permanent disturbance in the microbial community structure. In this experiment, we aimed to study the effect that kefir could cause in both fermentation models and whether this effect was due directly to the fermentation of kefir inoculate or indirectly by a change in the resilience of the microbiota; permutational analysis of the gathered data indicated a distinctive response in both fermentative models to both direct or indirect effects (**Figure 5**), and a significant effect of the addition of kefir on the fermentation pattern. However, presence of kefir did not indirectly change in the activity of microbial population over the fermented substrate (**Figure 5B**, *P*_treatment_ = 0.389), but induced a more variable response in the hindgut rather than the foregut model (**Figure 6**). Although both models effectively reacted to the new conditions by adapting to the disturbance induced by kefir, the variability of that response, measured by the dispersion of that response throughout the different samples was not the same, suggesting that foregut model is more robust than the hindgut one, which was more variable in such response. Thus, these results suggest that hindgut model is less stable than the foregut one, and hence more prone to experiment disturbances that could be maintained over a longer period of time. Moreover, the dispersion of the samples with respect with their centroid (**Figure 6B**), indicated that the increase in the variability became more significant when the indirect influence of kefir was studied.

## Conclusion

The addition of kefir boosted fermentation in the gut, stimulating VFA production, shifting it toward propionic fermentation and increasing the maximum potential of fermentation in both fermentation models. Heat processing techniques like pasteurization or autoclaving influenced the way the kefir is fermented and reacts with the present microbiota. In terms of comparison between both models, the foregut model seems to be less affected by the inclusion of Kefir than the hindgut model, in terms of variability in the response; moreover the hindgut model appeared to be a less stable model than the foregut model in the way it reacted indirectly to the addition of different types of kefir.

The combination of indirect approaches and multivariate analysis can be a valuable and affordable way to explore stability of complex microbial environments.

## Author Contributions

Conceived and designed the experiments: GdF, CN. Performed the experiments: GdF, EJ. Analyzed the data: GdF. Contributed reagents/materials/analysis tools: CN, SJ. Wrote the paper: GdF, CN. Reviewed the paper: GdF, CN, SJ, EJ.

## Conflict of Interest Statement

The authors declare that the research was conducted in the absence of any commercial or financial relationships that could be construed as a potential conflict of interest.
