# Supplementary material for: Functional Resilience and Response to a Dietary Additive (Kefir) in Models of Foregut and Hindgut Microbial Fermentation In Vitro
Source: Front Microbiol. 2017 Jun 28;8:1194. doi: 10.3389/fmicb.2017.01194 (PMC5487516; doi:10.3389/fmicb.2017.01194)
Supplement: Supplementary file 1 [file Table_1.DOCX]

Table S1: Chemical composition of experimental diets used in both foregut (RUM) and hindgut (HOR) fermentation models.

| Ingredient | | RUM | HOR | |
| --- | --- | --- | --- | --- |
| Dry matter (g/Kg FM) | 918.43 | | 938.61 |  |
| Organic matter (g/Kg DM) | 909.23 | | 901.7 |  |
| Crude protein (g/Kg DM) | 158.42 | | 147.76 |  |
| Neutral detergent fibre (g/Kg DM) | 422.58 | | 599.51 |  |
| Acid detergent fibre(g/Kg DM) | 270.4 | | 373.96 |  |
| Ether extract (g/Kg DM) | 13.63 | | 16.26 |  |
| Non-fibre carbohydrates (g/Kg DM) | 314.6 | | 138.17 |  |
