# Supplementary material for: Functional Resilience and Response to a Dietary Additive (Kefir) in Models of Foregut and Hindgut Microbial Fermentation In Vitro
Source: Front Microbiol. 2017 Jun 28;8:1194. doi: 10.3389/fmicb.2017.01194 (PMC5487516; doi:10.3389/fmicb.2017.01194)
Supplement: Supplementary file 2 [file Table_2.DOCX]

Table S2: Branched-chain volatile fatty acids production (Mm) after 24 h of incubation of a mixed diet in a hindgut model of rumen fermentation incubated with kefir (CTR), unaltered kefir (KEF), autoclaved kefir (AUT) or pasteurised kefir (PAS) after 24 h of incubation. SED means Standard error of the difference between means (N=4).

|  | CTR | KEF | AUT | PAS | SED | Significance |
| --- | --- | --- | --- | --- | --- | --- |
| Iso-butyric | 0.61 | 0.73 | 0.71 | 0.73 | 0.015 | *** |
| Iso-valeric | 0.89 | 1.20 | 1.14 | 1.22 | 0.017 | *** |
| N-valeric | 1.22 | 1.61 | 1.53 | 1.65 | 0.021 | *** |
| N-caproic | 0.45 | 0.74 | 0.68 | 0.75 | 0.011 | *** |

T means 0.1>P>0.05;* means P<0.05; ** means P<0.01; *** means P<0.001; NS means P>0.1
