# Supplementary material for: Functional Resilience and Response to a Dietary Additive (Kefir) in Models of Foregut and Hindgut Microbial Fermentation In Vitro
Source: Front Microbiol. 2017 Jun 28;8:1194. doi: 10.3389/fmicb.2017.01194 (PMC5487516; doi:10.3389/fmicb.2017.01194)
Supplement: Supplementary file 3 [file Table_3.DOCX]

Table S3: Branched-chain volatile fatty acids production (Mm) after 24 h of incubation of a mixed diet in a hindgut model of equine fermentation incubated with kefir (CTR), unaltered kefir (KEF), autoclaved kefir (AUT) or pasteurised kefir (PAS) after 24 h of incubation. SED means Standard error of the difference between means (N=4).

|  | CTR | KEF | AUT | PAS | SED | Significance |
| --- | --- | --- | --- | --- | --- | --- |
| Iso-butyric | 0.70 | 0.81 | 0.82 | 0.89 | 0.051 | * |
| Iso-valeric | 1.07 | 1.31 | 1.24 | 1.35 | 0.019 | *** |
| N-valeric | 0.34 | 0.31 | 0.33 | 0.30 | 0.027 | NS |
| N-caproic | 0.01 | 0.08 | 0.06 | 0.09 | 0.004 | *** |

T means 0.1>P>0.05;* means P<0.05; ** means P<0.01; *** means P<0.001; NS means P>0.1
