# Supplementary material for: Functional Resilience and Response to a Dietary Additive (Kefir) in Models of Foregut and Hindgut Microbial Fermentation In Vitro
Source: Front Microbiol. 2017 Jun 28;8:1194. doi: 10.3389/fmicb.2017.01194 (PMC5487516; doi:10.3389/fmicb.2017.01194)
Supplement: Supplementary file 4 [file Table_4.DOCX]

Table S4: Branched-chain volatile fatty acids production (Mm) after 24 h of incubation of a mixed diet in a foregut model of rumen fermentation incubated with kefir (CTRB), unaltered kefir (KEFB), autoclaved kefir (AUTB) or pasteurised kefir (PASB) after subtracting their respective blanks (containing only kefir), after 24 h of incubation. SED means Standard error of the difference between means (N=4).

|  | CTRB | KEFB | AUTB | PASB | SED | Significance |
| --- | --- | --- | --- | --- | --- | --- |
| Iso-butyric | 0.16 | 0.18 | 0.21 | 0.19 | 0.061 | NS |
| Iso-valeric | 0.02 | 0.12 | 0.09 | 0.04 | 0.032 | * |
| N-valeric | 0.03 | 0.02 | 0.00 | -0.02 | 0.036 | NS |
| N-caproic | - | - | - | - | - | - |

T means 0.1>P>0.05;* means P<0.05; ** means P<0.01; *** means P<0.001; NS means P>0.1
